# Estimating Infection Attack Rates and Severity in Real Time during an Influenza Pandemic: Analysis of Serial Cross-Sectional Serologic Surveillance Data

**DOI:** 10.1371/journal.pmed.1001103

**Published:** 2011-10-04

**Authors:** Joseph T. Wu, Andrew Ho, Edward S. K. Ma, Cheuk Kwong Lee, Daniel K. W. Chu, Po-Lai Ho, Ivan F. N. Hung, Lai Ming Ho, Che Kit Lin, Thomas Tsang, Su-Vui Lo, Yu-Lung Lau, Gabriel M. Leung, Benjamin J. Cowling, J. S. Malik Peiris

**Affiliations:** 1 Department of Community Medicine and School of Public Health, Li Ka Shing Faculty of Medicine, The University of Hong Kong, Hong Kong Special Administrative Region, People's Republic of China; 2 Department of Microbiology, Li Ka Shing Faculty of Medicine, The University of Hong Kong, Hong Kong Special Administrative Region, People's Republic of China; 3 Hong Kong Red Cross Blood Transfusion Service, Hospital Authority, Hong Kong Special Administrative Region, People's Republic of China; 4 Department of Medicine, Li Ka Shing Faculty of Medicine, The University of Hong Kong, Hong Kong Special Administrative Region, People's Republic of China; 5 Centre for Health Protection, Department of Health, Government of the Hong Kong Special Administrative Region, People's Republic of China; 6 Hospital Authority, Hong Kong Special Administrative Region, People's Republic of China; 7 Food and Health Bureau, Government of the Hong Kong Special Administrative Region, People's Republic of China; 8 Department of Paediatrics and Adolescent Medicine, Li Ka Shing Faculty of Medicine, The University of Hong Kong, Hong Kong Special Administrative Region, People's Republic of China; 9 HKU-Pasteur Research Center, Hong Kong Special Administrative Region, People's Republic of China; National Institutes of Health, United States of America

## Abstract

**Background:**

In an emerging influenza pandemic, estimating severity (the probability of a severe outcome, such as hospitalization, if infected) is a public health priority. As many influenza infections are subclinical, sero-surveillance is needed to allow reliable real-time estimates of infection attack rate (IAR) and severity.

**Methods and Findings:**

We tested 14,766 sera collected during the first wave of the 2009 pandemic in Hong Kong using viral microneutralization. We estimated IAR and infection-hospitalization probability (IHP) from the serial cross-sectional serologic data and hospitalization data. Had our serologic data been available weekly in real time, we would have obtained reliable IHP estimates 1 wk after, 1–2 wk before, and 3 wk after epidemic peak for individuals aged 5–14 y, 15–29 y, and 30–59 y. The ratio of IAR to pre-existing seroprevalence, which decreased with age, was a major determinant for the timeliness of reliable estimates. If we began sero-surveillance 3 wk after community transmission was confirmed, with 150, 350, and 500 specimens per week for individuals aged 5–14 y, 15–19 y, and 20–29 y, respectively, we would have obtained reliable IHP estimates for these age groups 4 wk before the peak. For 30–59 y olds, even 800 specimens per week would not have generated reliable estimates until the peak because the ratio of IAR to pre-existing seroprevalence for this age group was low. The performance of serial cross-sectional sero-surveillance substantially deteriorates if test specificity is not near 100% or pre-existing seroprevalence is not near zero. These potential limitations could be mitigated by choosing a higher titer cutoff for seropositivity. If the epidemic doubling time is longer than 6 d, then serial cross-sectional sero-surveillance with 300 specimens per week would yield reliable estimates when IAR reaches around 6%–10%.

**Conclusions:**

Serial cross-sectional serologic data together with clinical surveillance data can allow reliable real-time estimates of IAR and severity in an emerging pandemic. Sero-surveillance for pandemics should be considered.

*Please see later in the article for the Editors' Summary*

## Abstract 2

This study reports that using serological data coupled with clinical surveillance data can provide real-time estimates of the infection attack rates and severity in an emerging influenza pandemic.

## Editors' Summary

**Background:**

Every winter, millions of people catch influenza—a viral infection of the airways—and about half a million die as a result. These seasonal epidemics occur because small but frequent changes in the influenza virus mean that the immune response produced by infection with one year's virus provides only partial protection against the next year's virus. Occasionally, however, a very different influenza virus emerges to which people have virtually no immunity. Such viruses can start global epidemics (pandemics) and kill millions of people. The most recent influenza pandemic began in March 2009 in Mexico, when the first case of influenza caused by a new virus called pandemic A/H1N1 2009 (pdmH1N1) occurred. The virus spread rapidly despite strenuous efforts by national and international public health agencies to contain it, and on 11 June 2009, the World Health Organization (WHO) declared that an influenza pandemic was underway. By the time WHO announced that the pandemic was over (10 August 2010), pdmH1N1 had killed more than 18,000 people.

**Why Was This Study Done?:**

Early in the 2009 influenza pandemic, as in any emerging pandemic, reliable estimates of pdmH1N1's transmissibility (how easily it spreads between people) and severity (the proportion of infected people who needed hospital treatment) were urgently needed to help public health officials plan their response to the pandemic and advise the public about the threat to their health. Because infection with an influenza virus does not always make people ill, the only way to determine the true size and severity of an influenza outbreak is to monitor the occurrence of antibodies (proteins made by the immune system in response to infections) to the influenza virus in the population—so-called serologic surveillance. In this study, the researchers developed a method that uses serologic data to provide real-time estimates of the infection attack rate (IAR; the cumulative occurrence of new infections in a population) and the infection-hospitalization probability (IHP; the proportion of affected individuals that needs to be hospitalized) during an influenza pandemic.

**What Did the Researchers Do and Find?:**

The researchers tested nearly 15,000 serum samples collected in Hong Kong during the first wave of the 2009 pandemic for antibodies to pdmH1N1 and then used a mathematical approach called convolution to estimate IAR and IHP from these serologic data and hospitalization data. They report that if the serological data had been available weekly in real time, they would have been able to obtain reliable estimates of IAR and IHP by one week after, one to two weeks before, and three weeks after the pandemic peak for 5–14 year olds, 15–29 year olds, and 30–59 year olds, respectively. If serologic surveillance had begun three weeks after confirmation of community transmission of pdmH1N1, sample sizes of 150, 350, and 500 specimens per week from 5–14 year olds, 15–19 year olds, and 20–29 year olds, respectively, would have been sufficient to obtain reliable IAR and IHP estimates four weeks before the pandemic peak. However, for 30–59 year olds, even 800 specimens per week would not have generated reliable estimates because of pre-existing antibodies to an H1N1 virus in this age group. Finally, computer simulations of future pandemics indicate that serologic surveillance with 300 serum specimens per week would yield reliable estimates of IAR and IHP as soon as the true IAR reached about 6%.

**What Do These Findings Mean?:**

These findings suggest that serologic data together with clinical surveillance data could be used to provide reliable real-time estimates of IARs and severity in an emerging influenza pandemic. Although the number of samples needed to provide accurate estimates of IAR and IHP in real life may vary somewhat from those reported here because of limitations in the design of this study, these findings nevertheless suggest that the level of testing capacity needed to provide real-time estimates of IAR and IHP during an emerging influenza pandemic should be logistically feasible for most developed countries. Moreover, collection of serologic surveillance data from any major city affected early in an epidemic could potentially provide information of global relevance for public health. Thus, the researchers conclude, serologic monitoring should be included in future plans for influenza pandemic preparedness and response and in planning for other pandemics.

**Additional Information:**

Please access these websites via the online version of this summary at http://dx.doi.org/10.1371/journal.pmed.1001103.

- A recent *PLoS Medicine* Research Article by Riley et al. provides further information on patterns of infection with the pdmH1N1 virus
- The Hong Kong Centre for Health Protection provides information on pandemic H1N1 influenza
- The US Centers for Disease Control and Prevention provides information about influenza for patients and professionals, including specific information on H1N1 influenza
- Flu.gov, a US government website, provides access to information on seasonal, pandemic, and H1N1 influenza
- WHO provides information on seasonal influenza and has information on the global response to H1N1 influenza (in several languages)
- The UK Health Protection Agency provides information on pandemic influenza and on H1N1 influenza
- More information for patients about H1N1 influenza is available through Choices, an information resource provided by the UK National Health Service

## Introduction

One of the lessons learned from the 2009 H1N1 influenza (pdmH1N1) pandemic was the need for rapid and reliable estimates of transmissibility and severity (the probability of severe outcomes, such as hospitalization and death, if infected) of the novel virus [1]. This is crucial for public health planning and for effective communication with the public. Early efforts were hampered by limited data [2], and while initial estimates of a basic reproductive number in the range of 1.2–1.6 were consistent with findings in other countries that were subsequently affected [3]–[5], the initial estimate of case-fatality probability of 0.4% now appears to be substantially overestimated [6],[7].

In June 2009, we established a comprehensive serologic survey of pdmH1N1 in Hong Kong. Facilitated by enhanced local laboratory capacity developed since the 2003 epidemic of severe acute respiratory syndrome, Hong Kong used extensive laboratory testing for pdmH1N1 among all hospitalizations with respiratory illness throughout the 2009 influenza pandemic. We previously reported pdmH1N1 infection attack rate (IAR) and severity estimates using only serologic data collected before and immediately after the first wave of the pandemic in Hong Kong [7]. A companion study used paired sera collected from a cohort (1) to estimate the IAR and severity profile of pdmH1N1 in Hong Kong and (2) to show that specimens collected around the peak of an epidemic from larger cohorts could have yielded more reliable severity estimates [8]. In this paper, we used all available serial cross-sectional serologic data to investigate how soon we would have obtained reliable estimates of IAR and infection-hospitalization probability (IHP) (the probability of hospitalization if infected) if these serologic data were available weekly in real-time as the epidemic unfolded. Having illustrated the principle of serial cross-sectional sero-surveillance for pdmH1N1, we then conducted extensive computer simulations to assess its expected performance and logistical requirements in future pandemics.

This study was organized as follows. First, we described a convolution-based method for real-time estimation of IAR and IHP from clinical surveillance and serial cross-sectional serologic data. The same method has been used to estimate incidence of pdmH1N1 in England [9],[10]. Next, we retrospectively applied this method to our pdmH1N1 hospitalization and serologic data to sequentially compute real-time estimates of IHP and IAR that would have been obtained as the epidemic unfolded. We then estimated the number of specimens that would have been required in order to obtain reliable estimates of IHP and IAR 3–4 wk before the epidemic peak. Finally, we conducted computer simulations with hypothetical pandemic scenarios to analyze how the performance of serial cross-sectional sero-surveillance depends on the characteristics of serologic testing (sensitivity, specificity, throughput, lead time, titer cutoff, pre-existing seroprevalence) and epidemic dynamics (basic reproductive number, generation time, natural history, antibody response kinetics). Our goal was to provide operational guidelines for implementing serial cross-sectional sero-surveillance in future pandemics of influenza and other infectious diseases.

## Methods

### Clinical Surveillance Data

Age-stratified data on the daily number of virologically confirmed outpatient consultations, hospitalizations, intensive care unit admissions, and deaths associated with pdmH1N1 from 29 April 2009 to 30 November 2009 were provided by the e-flu database of the Hong Kong Hospital Authority [11],[12]. Beginning May 2009, patients admitted with acute respiratory illnesses routinely underwent laboratory testing for pdmH1N1 virus, with laboratory results available typically within 24 h and notification to the central database typically within 1–2 d [7]. Local pdmH1N1 transmission was identified in mid-June, but containment efforts enforced until 29 June 2009 required all laboratory-confirmed cases to be hospitalized for isolation regardless of disease severity, and therefore only surveillance data from 30 June 2009 onwards were used in our analysis. In this study, we focused on estimating the IHP, which was defined as the probability that an infected case (not necessarily symptomatic) required hospitalization. In our earlier publication [7], we called this quantity case-hospitalization rate. Here, we revised the terminology to avoid confusion with the probability of hospitalization if infected with symptoms (e.g., [6]). We assumed that IHP was constant from 30 June 2009 onwards.

### Seroprevalence Data

Between 12 June 2009 and 30 June 2010, we tested 13,328 serum samples from blood donors (aged 16–59 y), 3,613 from hospital outpatients (aged 5–90 y), and 917 from participants of a community pediatric cohort study (aged 5–14 y). Further description of the study design and preliminary analyses of a subset of these sera collected before and immediately after the first wave of pdmH1N1 can be found in [7] and Text S1. Sera were tested for antibody responses to A/California/4/2009 (H1N1) by viral microneutralization (MN). Our definition of MN titer in our previous publication [7] and the current study is slightly different from the latest World Health Organization (WHO) recommendation published in 2011 [13]. We followed the previous convention in which MN titers were denoted by taking into account the final dilution resulting from mixing the serum dilution with the virus. The latest WHO manual for laboratory diagnosis recommends that the virus titer be denoted as the initial serum dilution alone [13]. In effect, our MN titers in [7] and the current study need to be halved when comparing them with those that follow the latest recommendation (e.g., those in Veguilla et al. [14], which we used to estimate the antibody response kinetics parameters for the current study; see below and Text S1 for details).

We defined pdmH1N1 seropositivity as an MN antibody titer of ≥1∶40 and pdmH1N1 seroprevalence as the proportion of individuals who were seropositive. The age-specific seroprevalence of the three groups of participants were largely similar across time during the first wave (Figure S1). Estimates of IAR among pdmH1N1 serology studies from different countries using different sampling schemes have been quite similar [7],[8],[15],[16]. To build a model for illustrating the principle of serial cross-sectional sero-surveillance, we aggregated the seroprevalence data from the three groups of participants, though we acknowledge that such aggregation is not generally well-justified in terms of representativeness. Specimens collected before 30 June 2009 were collectively used to estimate the seroprevalence on 30 June 2009. Serologic data between 30 June 2009 and 30 November 2009 were grouped into weekly batches, and the collection time of each batch was set to be the average collection time of its constituents (i.e., weighted by the number of samples each day). In summary, serologic data used in this study comprised 14,766 samples collected from 5–59 y olds before 30 November 2009.

### A Convolution-Based Method for Real-Time Estimation of IAR and Severity

**Figure 1:**
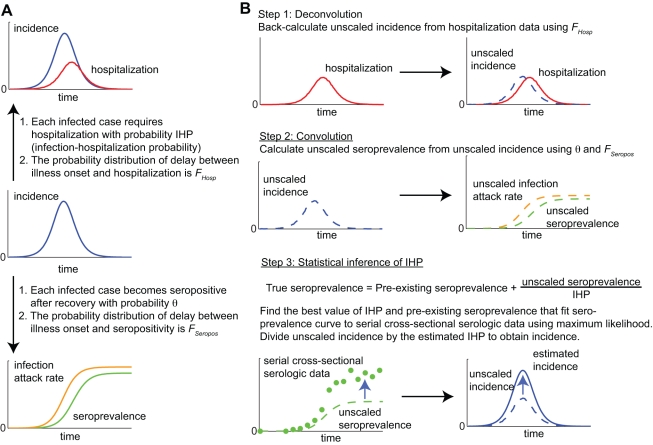
A schematic of the convolution-based method for real-time estimation of IHP and IAR from hospitalization and serial cross-sectional serologic data. (A) The hospitalization (top) and seroprevalence (bottom) curves are both delayed and scaled transformations of the incidence curve (middle). (B) By performing the reverse transformations, we can use hospitalization and seroprevalence data to reconstruct incidence and estimate IHP and IAR in real time.

We used a convolution-based method for obtaining real-time estimates of IHP and IAR from serial cross-sectional serologic data and hospitalization data. The same method has been used to estimate incidence of pdmH1N1 in England [9],[10]. A schematic of this method is shown in Figure 1. The method requires knowing (1) the cumulative distribution function of the time from illness onset to hospitalization *F*_Hosp_, (2) the cumulative distribution function of time from illness onset to seropositivity *F*_Seropos_, and (3) the proportion of infections that eventually became seropositive, θ. In principle, all these should be directly observable from pandemic surveillance. The basic algorithm of this method was as follows. At any time *t* during the epidemic: (1) Use *F*_Hosp_ to deconvolute daily hospitalizations *h*_0_,…,*h_t_* to obtain an unscaled incidence (daily number of infections) curve *a*_0_,…,*a_t_* [17]. If IHP is known, the true incidence curve is estimated by dividing *a*_0_,…,*a_t_* by IHP. This step can be skipped if the actual onset dates of hospitalized cases are known. (2) Use *F*_Seropos_ to construct an estimated seroprevalence curve *b*_0_,…,*b_t_* from the unscaled incidence curve *a*_0_,…,*a_t_*:
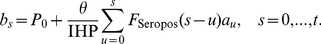
(1)where *P*_0_ is the true pre-pandemic seroprevalence. (3) Fit the estimated seroprevalence curve *b*_0_,…,*b_t_* to the serial cross-sectional serologic data by finding the values of IHP and *P*_0_ that maximize the following likelihood function:
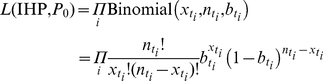
(2)where the product is over all times *t_i_*≤*t* for which cross-sectional serologic data are available, with each component being the (binomial) probability of getting 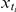
seropositives from testing 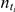 samples collected at time *t_i_* if the true seroprevalence was 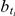. IAR can then be estimated by dividing the unscaled incidence curve by our maximum likelihood estimate (MLE) of IHP.

In this basic algorithm, sensitivity and specificity of serologic testing were assumed to be 100%. The method can be extended to incorporate imperfect sensitivity and specificity, temporal variation in IHP (e.g., weekend and seasonal effects) and different titer cutoffs for seropositivity. See Text S1 for the generalized algorithm that takes into account these factors. Note that sensitivity (specificity) here referred to the probability that the result of the serologic test was positive (negative) if the serum specimen was truly seropositive (seronegative), regardless of whether seropositivity was due to pre-existing cross-reactive antibodies or antibodies generated by recent pandemic infection. Therefore, our definitions of sensitivity and specificity were different from that in recent related publications on the performance of pdmH1N1 serologic assays in which sensitivity was defined as the probability of a positive serologic result among infected individuals and specificity the probability of a negative serologic result among uninfected individuals [14],[18].

### A Model for Retrospective Real-Time Estimation of pdmH1N1 IHP and IAR

**Figure 2:**
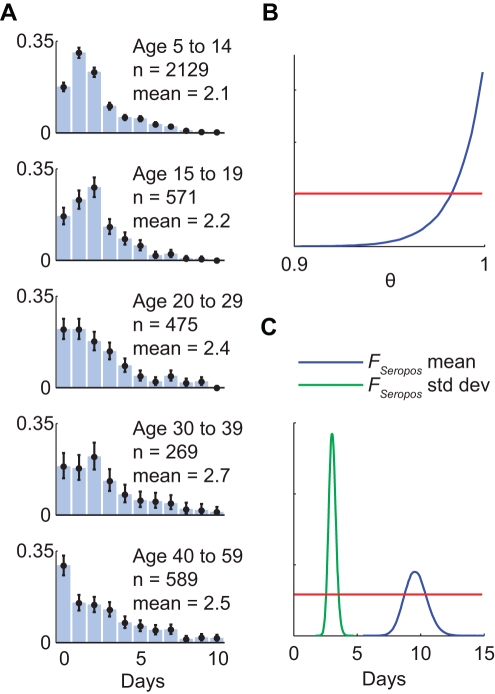
Time delay from illness onset to hospitalization, proportion of infected cases who reached seropositivity, and time delay from illness onset to seropositivity. (A) Probability density functions of the age-specific delay between illness onset and hospitalization as observed in the e-flu database surveillance data. Black bars indicate 95% confidence intervals. (B and C) Statistical analysis of published data on the kinetics of pdmH1N1 antibody response among laboratory-confirmed cases in the US [14]. Posterior distributions were obtained using Markov chain Monte Carlo method with non-informative priors (red lines); see Text S1 for details. (B) The posterior distribution of the proportion of laboratory-confirmed cases who eventually developed MN titer ≥1∶40. (C) The posterior distributions of the mean and standard deviation of the delay between illness onset and seropositivity assuming that the delay distribution *F*_Seropos_ was Erlang-10.

When retrospectively applying the convolution-based method to our pdmH1N1 data, we made the following model specifications. (1) IAR and IHP were estimated for the following age groups for ease of comparison with our previous study [7]: 5–14, 15–19, 20–29, 30–39, and 40–59 y. (2) Sensitivity and specificity were 100% for serologic testing for MN titer ≥1∶40. (3) Serologic results for each batch of specimens were available 3 d after the last sample of that batch was collected; *t_i_* in the likelihood function of Step 3 in the basic algorithm was defined to be the average collection time of the specimens contained in the *i*th batch. (4) For simplicity, we ignored the delay between infection and illness onset (around 1 d). Incorporating this delay would essentially shift the estimated incidence curve to the left by the length of the delay. (5) The upper-bound of age-specific IHP at time *t* was the cumulative number of hospitalizations divided by the cumulative number of confirmed cases up to time *t* for that age group. Similarly, the lower-bound was the cumulative number of hospitalizations divided by the size of that age group. (6) The cumulative distribution function of the time from illness onset to hospitalization *F*_Hosp_ was based on those hospitalized cases whose onset dates were available in our clinical surveillance data (Figure 2A). (7) The proportion of infected individuals who eventually became seropositive θ and the cumulative distribution function of the time from illness onset to seropositivity *F*_Seropos_ were estimated using published data on the kinetics of antibody response among laboratory-confirmed pdmH1N1 cases in the United States [14]. To simplify our analysis, we assumed that *F*_Seropos_ was an Erlang-10 distribution with mean μ_Seropos_ and constructed a likelihood *L*_A_(θ, μ_Seropos_) for these antibody response data (results were almost identical when Erlang-5, -20, or -40 was used instead; see Text S1 for details). The resulting MLEs were θ = 1 and μ_Seropos_ = 9.6 d. However, given the modest sample size of this study, these estimates were associated with significant uncertainty (Figure 2B and 2C). To incorporate such uncertainty into our real-time estimates of IAR and IHP, we modified the convolution-based method to estimate IHP, *P*_0_, θ, and *F*_Seropos_ simultaneously by redefining the likelihood as the product of *L*(IHP, *P*_0_) in Step 3 above and *L*_A_(θ, μ_Seropos_). Our premise was that antibody response data of similar sample size and precisions could have been obtained in real-time during the early phase of the pandemic from serologic follow-up of the first virologically confirmed cases [9]. We defined the full model as the estimates of IHP, *P*_0_, θ, and *F*_Seropos_ obtained from the full set of hospitalization and serial cross-sectional serologic data (i.e., up to 30 November 2009).

In this model, our IAR estimate would be accurate if and only if our IHP estimate was accurate. As such, for conciseness, we focus on the latter when presenting our results. When evaluating the reliability of sequential real-time estimates of IHP, we used the full model as the reference for comparison, i.e., we assumed that the full model gave accurate estimates of the true IHP. In this context, we regarded a real-time IHP estimate as reliable if (1) its MLE did not differ from the MLE in the full model by more than 50% and (2) its interquartile range (IQR) was less than three times its MLE.

### Serial Cross-Sectional Sero-Surveillance for Future Pandemics

To assess the logistical requirements and expected performance of serial cross-sectional sero-surveillance for future pandemics, we first estimated the number of specimens that would have allowed reliable estimates of IHP for pdmH1N1 by mid-August 2009 (4 wk before the epidemic peak), assuming that the incidence and seroprevalence curves in the full model were accurate. We simulated 300 stochastic realizations of serial cross-sectional sero-surveillance in which (1) *m* pre-pandemic specimens were used to estimate seroprevalence on 30 June 2009 and (2) *m* specimens were collected and tested every week starting in the fourth week of July 2009 (3 wk after community transmission was confirmed). Sequential real-time estimates of IHP were then computed using the convolution-based method. We searched for the smallest value of *m* for each age group that would yield reliable estimates of IHP by mid-August.

Next, we conducted simulations with hypothetical epidemic scenarios in order to analyze the general behavior of serial cross-sectional sero-surveillance. We first considered susceptible-infected-removed epidemic dynamics with a basic reproductive number of *R*_0_ = 1.4, mean generation time of *T*_g_ = 2.5 d, IHP = 0.5%, and Erlang-3 probability distribution for the infectious duration with mean 2*T*_g_ *w*/(1 + *w*) = 3.75 d, where *w* = 3 is the number of Erlang stages [19],[20]. We assumed that the probability distribution *F*_Hosp_ was the same as that in our pdmH1N1 model (Figure 2A). We assumed that 100 sera with collection times uniformly distributed between 1 and 28 d after symptom onset were available for estimating θ and *F*_Seropos_ (as in model specification number 7 for pdmH1N1 above; see Text S1 for details). We simulated serial cross-sectional sero-surveillance with 300 serum samples per week starting 28 d after 50 infections were seeded in a population of 1 million. The 28 d of delay after seeding was meant to reflect the time needed to develop a reliable serologic assay and to set up the sero-surveillance operations. We simulated the following scenarios to study the effect of sensitivity and specificity of serologic testing, pre-existing seroprevalence, and alternative titer cutoff for seropositivity: (A) 100% sensitivity, 100% specificity, no pre-existing seroprevalence, θ = 1, and *F*_Seropos_ of Erlang-10 with mean 9.6 d (i.e., same as the MLEs for the US antibody response data); (B) same as scenario A but with 80% sensitivity; (C) same as scenario A but with 95% specificity; (D) same as scenario A but with 5% pre-existing seroprevalence; (E) same as scenario A but with a higher titer cutoff for seropositivity such that θ = 0.6 and the mean of *F*_Seropos_ increased by 50%.

Finally, to investigate the dependence on epidemic dynamics, we simulated serial cross-sectional sero-surveillance in 100 epidemic scenarios that were randomly generated using Latin-hypercube sampling of the following parameter space: *R*_0_ between 1.2 and 2; *T*_g_ between 2 and 4 d; IHP between 0.1% and 3%; the probability distribution of infectious duration Erlang-*k*, *k* = 1, …,5; population size between 250,000 and 2.5 million; θ between 0.6 and 1; and *F*_Seropos_ gamma with mean between 6 and 16 d and coefficient of variation (standard deviation divided by mean) between 0.1 and 0.6. Both the mean and standard deviation of *F*_Seropos_ were included for statistical inference, i.e., this was a relaxation of our previous Erlang-10 assumption for *F*_Seropos_.

For each of these epidemic scenarios, we compared the performance of sero-surveillance under the following operational conditions: (1) sero-surveillance begun 28 d after seeding, with 150, 300, and 450 specimens per week; (2) sero-surveillance begun 14, 28, and 42 d after seeding, with 300 specimens per week.

### Ethics Committee Approval

All study protocols were approved by the Institutional Review Board of the University of Hong Kong/Hospital Authority Hong Kong West Cluster.

## Results

### Seroprevalence, IHP, and Final IAR in the Full Model

**Figure 3:**
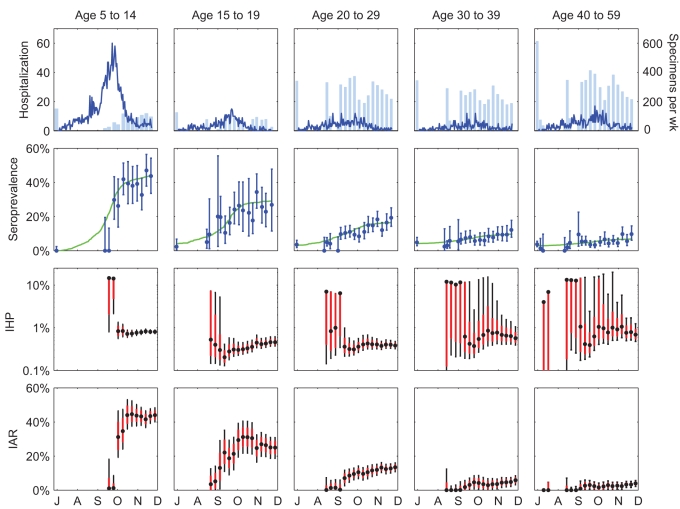
Hospitalization data, serial cross-sectional serologic data, and sequential real-time estimates of IHP and IAR for pdmH1N1 in Hong Kong. Each tick on the *x*-axis indicates the first day of the months July through December 2009. The first row shows the daily number of hospitalizations (blue lines) and the number of serum specimens (light blue bars) tested before 30 June 2009 (left-most bar in each graph) and in subsequent weeks. The second row shows the serial cross-sectional serologic data (blue circles indicate MLEs and bars indicate 95% confidence intervals of seroprevalence in each cross-section) and the seroprevalence curves in the full model (green lines). The third and fourth rows show the sequential real-time estimates of IHP and IAR, respectively (black circles for MLEs, black bars for 95% confidence intervals, and red boxes for IQRs). Estimates were sequentially updated upon each new cross-section of serologic data. Each batch of serologic data was assumed to be available 3 d after the last sample of that batch was collected.

The age-specific seroprevalence curves in the full model provided a reasonably good fit to the serial cross-sectional serologic data (Figure 3) except for the first 2 wk of September for 5–14 y olds. For this period, seroprevalence in the full model was substantially higher than the proportion of seropositive sera in the data. This discrepancy was likely due to the small number of serum specimens available in these 2 wk (17 and 26). Age-specific IHP and final IAR in the full model were mostly similar to our previous estimates, which were based on only pre- and post-first-wave sera (Table 1). The largest discrepancy was that the final IAR for 15–19 y olds in the full model was 9% higher than our previous estimate. However, this was expected because of the inclusion of outpatient sera in the full model but not in our previous estimates. As noted in our previous study [7] and Text S1, the post-first-wave seroprevalence of outpatients was substantially higher than that of blood donors for this age group, hence the higher final IAR in the full model.

**Table 1:** Comparison of the estimates of IHP and IAR in the full model with those from our previous study, which used only the pre- and post-first-wave sera [7].

Age Group	IHP		IAR	
	Full Model	Pre- and Post-First-Wave Sera [7]	Full Model	Pre- and Post-First-Wave Sera [7]
5–14 y	0.80% (0.73%–0.88%)	0.84% (0.76%–0.97%)	44.0% (40.8%–47.3%)	43.4% (37.9%–47.6%)
15–19 y	0.46% (0.37%–0.62%)	0.77% (0.53%–1.50%)	25.0% (19.7%–30.1%)	15.8% (8.2%–22.1%)
20–29 y	0.39% (0.32%–0.48%)	0.47% (0.37%–0.66%)	13.4% (11.3%–15.6%)	11.8% (8.4%–14.7%)
30–39 y	0.57% (0.39%–1.06%)	0.80% (0.45%–3.66%)	5.8% (3.6%–8.1%)	4.3% (0.9%–7.5%)
40–59 y	0.69% (0.48%–1.24%)	0.61% (0.38%–1.07%)	3.9% (2.5%–5.4%)	5.0% (2.7%–7.4%)

Full model results are MLE (95% confidence interval); pre- and post-first-wave sera results are posterior mode (95% credible interval).

### Retrospective Sequential Real-Time Estimates of IHP and IAR for pdmH1N1

Had our serologic data been available weekly in real time, reliable estimates of IHP would have been available in early October 2009 for 5–14 y olds, early September 2009 for 15–29 y olds, and mid-October 2009 for 30–59 y olds (Figure 3). These time points corresponded to 1 wk after, 1–2 wk before, and 3 wk after the epidemic peak. For the 5–14 y olds, reliable estimate of IHP would not have been available before the peak because the number of serum specimens was small and collection of sera did not begin until 3 wk before the peak for this age group. For the 30–59 y olds, reliable estimate of IHP would not have been available until the first wave was almost over because the final IAR was comparable in magnitude to the pre-existing seroprevalence for this age group. That is, the signal-to-background ratio was small, which required a larger number of sera (relative to the average of 200–300 specimens per week in our study; see Figure 3) in order to accurately detect the increase in seroprevalence generated by pandemic infections (see below for further analysis and discussions). The sequential real-time estimates of IHP exhibited the following patterns: (1) the MLE zoomed to the correct order of magnitude upon the first cross-section of serologic data for which seroprevalence was apparently above pre-pandemic level, and (2) the confidence intervals widened upon each cross-section of serologic data for which seroprevalence was lower than the most up-to-date estimate in the model, e.g., because of statistical noise associated with sampling.

Had we begun weekly sero-surveillance in the fourth week of July 2009, we would have needed around 150, 350, and 500 specimens per week for 5–14 y olds, 15–19 y olds, and 20–29 y olds in order to obtain reliable estimates of IHP for these age groups by mid-August 2009 (Figure S2). For the 30–59 y olds, even a prohibitively large sample size of 800 per week would not have provided reliable estimates of IHP until mid- to late September 2009 because of the low ratio of IAR to pre-existing seroprevalence for these age groups.

### Serial Cross-Sectional Sero-Surveillance for Future Pandemics

**Figure 4:**
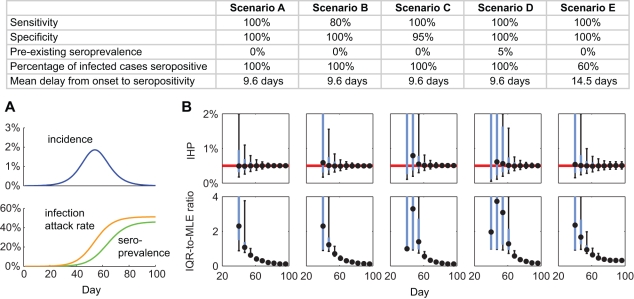
Serial cross-sectional sero-surveillance for future pandemics. (A) Simulated incidence, IAR, and seroprevalence using a susceptible-infected-removed model with basic reproductive number *R*_0_ = 1.4 and mean generation time *T*_g_ = 2.5 d. (B) Performance of serial cross-sectional sero-surveillance for the simulated epidemic in five scenarios (across the columns; see the table for the scenario descriptions). For each scenario, we simulated 500 stochastic realizations of sero-surveillance with 300 serologic samples per week starting 28 d after seeding. The top row of graphs shows the frequency distributions of sequential IHP MLEs, and the bottom row of graphs shows the frequency distribution of the sequential IQR-to-MLE ratios. In the top panels, the red lines indicate the true value of IHP = 0.5%. In all panels, black circles indicate the median, while blue boxes and black vertical bars indicate IQRs and 95% confidence intervals of the ordinate, respectively.

In the simulated base case (Figure 4, scenario A), serial cross-sectional sero-surveillance with 300 specimens per week yielded reliable estimates of IHP when the true seroprevalence was around 1%. With 100% of infected cases becoming seropositive 9.6 d after illness onset on average (Figure 2B and 2C), IAR was around 6% when seroprevalence was around 1%. This correspondence between IAR and seroprevalence was robust across epidemic model structure and parameter values (see Text S1 and Figure S3). The performance of serial cross-sectional sero-surveillance was largely unaffected even when the sensitivity of serologic testing was only 80% (Figure 4, scenario B). However, the performance substantially deteriorated if the specificity of serologic testing dropped from 100% to 95% (Figure 4, scenario C) or pre-existing seroprevalence increased from 0% to 5% (Figure 4, scenario D). This was because reliable estimation of IHP was mainly limited by how soon we could accurately detect an increase in seroprevalence generated by pandemic infections. When this signal was weak (i.e., during the early pandemic stage), accurate detection would be difficult when test specificity was low (i.e., with false positives decreasing the signal-to-noise ratio) or when pre-existing seroprevalence was not close to zero (i.e., the signal-to-background ratio was small). These limitations could be mitigated by increasing the titer cutoff for seropositivity. For example, serologic follow-up of 881 and 79 virologically confirmed pdmH1N1 cases in Hong Kong and the US found that around 57% and 94% of cases developed MN titer ≥1∶80 [14],[21]. With the seropositivity cutoff set to MN titer 1∶40 and 1∶80, the pre-existing seroprevalence in our serosurvey was 3%–5% and <0.2%, respectively [7]. Increasing the cutoff for seropositivity at the expense of decreasing the proportion of infected cases seropositive (θ) from 1 to 0.6 and increasing the mean delay from illness onset to seropositivity (the mean of *F*_Seropos_) by 50% from 9.6 d to 14.5 d would only slightly delay the timeliness of accurate estimates of IHP (Figure 4, scenario E).

**Figure 5:**
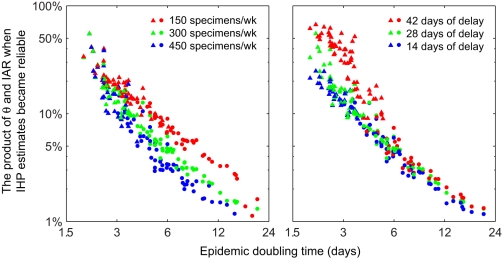
Performance of serial cross-sectional sero-surveillance in 100 randomly generated epidemic scenarios. All *x*- and *y*-axes are in log-scale. For each scenario, 200 stochastic realizations of serial cross-sectional sero-surveillance were simulated. (A) Sero-surveillance begun 28 d after epidemic seeding with 150 (red), 300 (green), and 450 (blue) specimens per week. (B) Sero-surveillance begun 42 (red), 28 (green), and 14 (blue) d after epidemic seeding with 300 specimens per week. Points represent the mean (among the 200 realizations) of θ × IAR at the threshold where a reliable estimate of IHP was obtained (defined as the stage of epidemic after which [1] all subsequent MLEs of IHP were within 50% of the true value and [2] all subsequent IQR-to-MLE ratios were less than three) in a given epidemic scenario; circles and triangles indicate that the reliable IHP estimate was obtained before and after the epidemic peak, respectively.

The performance of serial cross-sectional sero-surveillance depended on epidemic dynamics mostly via the epidemic doubling time (Figure 5). In general, if the epidemic doubling time was longer than 6 d, serial cross-sectional sero-surveillance with 300 serum specimens per week provided accurate estimates of IHP when θ × IAR reached around 6%. In this range of doubling time, the performance of sero-surveillance was largely similar when the delay between the start of sero-surveillance and epidemic seeding varied from 14 to 42 d. Given that the average delay from illness onset to seropositivity was around 9.6 d, it would be impossible for serial cross-sectional sero-surveillance to yield accurate estimates of IHP during the nascent stage of the epidemic if the epidemic doubling time was very short (Figure 5). The public health need for early severity estimates to inform situational awareness and pandemic response thus further highlights the importance of aggressive mitigation measures to slow the spread of disease during the early stages of a pandemic.

## Discussion

Our results suggest that had our serial cross-sectional serologic data been available weekly in real time during the 2009 influenza pandemic, reliable estimates of IAR and IHP could have been obtained 1 wk after, 1–2 wk before, and 3 wk after the epidemic peak for 5–14 y olds, 15–29 y olds, and 30–59 y olds, respectively. The ratio of IAR to pre-existing seroprevalence (the signal-to-background ratio), which decreased with age for pdmH1N1 in 2009, was a major determinant of the timeliness of reliable estimates. The 2009 pandemic provided a particular challenge from the point of view of serologic interpretation because it was caused by a virus subtype that was previously endemic in humans. This led to the presence of serologic cross-reactivity and therefore significant pre-existing seroprevalence at MN titer ≥1∶40, especially in the older age groups, hence the lack of timeliness of reliable IHP estimates in our retrospective analysis. This would have been much less of a problem with the pandemics of 1957 (H2N2) and 1968 (H3N2) or a future pandemic of H5N1. With H9N2 viruses, the challenge posed by serologic cross-reactions may be comparable to that with pdmH1N1 because a proportion of individuals born before 1968 appear to have cross-reactive antibodies [22]. Our results suggest that for serial cross-sectional sero-surveillance to yield timely and accurate estimates of IAR and severity, pre-existing seroprevalence needs be adjusted to near zero by choosing a sufficiently high titer cutoff for seropositivity. Given our limited serologic testing capacity, we only screened our specimens at MN titers of ≥1∶40 and ≥1∶20, without determining the exact antibody titer for each specimen. The performance of serial cross-sectional sero-surveillance might be enhanced if exact titers were available and incorporated into the real-time estimation of IHP. If hemagglutination inhibition rather than the more labor intensive MN tests were used (which may be feasible with some pandemic viruses), the logistical feasibility and performance of sero-surveillance may be further enhanced, although for pdmH1N1 the MN test was more sensitive and specific to confirmed infection [23]. Automation of serologic assays may increase feasibility of large-scale serology in the future.

The 2009 influenza pandemic highlighted the need for improved methods of rapid, reliable assessment of transmissibility and severity for an unfolding infectious disease outbreak. The Fineberg et al. [24] report on the performance of WHO during the pandemic highlighted the lack of “a consistent, measurable and understandable depiction of severity” as one of the shortcomings of the response in 2009, and called for proper timely assessment of severity to guide public health response. Real-time transmission modeling methods have previously been devised to estimate IAR and severity based on clinical surveillance data without the use of serologic data [25],[26]. Their performance depends on the reliability of the underlying transmission model, e.g., assumptions and data regarding contact patterns between age groups, medical consultation rates, and pre-existing immunity. In this study, we showed that serial cross-sectional sero-surveillance could complement these methods to allow timely and accurate real-time estimates of IAR and severity.

While the ideal sero-surveillance study would draw from a random sample of the population of interest, in practice this is unlikely to be feasible. In our study there was good agreement between specimens collected from blood donors, hospital outpatients, and community participants. A companion community-based cohort study with paired serologic data in Hong Kong also gave similar seroprevalence estimates [8]. Our study and other similar serologic studies [16],[27]–[29] have demonstrated that sero-surveillance is feasible and that the resulting information could provide invaluable data for accurate and timely estimation of population attack rates and disease severity. However, sero-surveillance does require substantial laboratory infrastructure and resources, and during a pandemic there may be competing concerns for laboratory services such as diagnostic testing and vaccine development. As in our case, involvement of academic research centers, which are less likely to be under pressure to provide front-line diagnostic services, may provide a feasible solution. The total cost of our serologic study was around 1% of the amount that Hong Kong spent on purchasing pdmH1N1 vaccines, whereas the information provided by our study has been instrumental in informing pandemic situational awareness and decisions for prioritizing vaccine target groups in Hong Kong.

In addition to having a reliable serologic assay, serologic follow-up of laboratory-confirmed cases needs to be conducted as early as possible during a pandemic in order to collect acute- and convalescence-phase sera for characterizing the kinetics of antibody response against the pandemic virus (θ and *F*_Seropos_) [14]. Kinetics of antibody response may be strain-specific. For example, Buchy et al. [30] analyzed 44 sera from 11 patients with H5N1 disease and found that no neutralizing antibodies were detected during the first week after disease onset, while 70% and 80% had MN titer ≥1∶80 2 and 3 wk after disease onset. Togo et al. [31] analyzed sera from seven individuals who were experimentally challenged with the A_2_/Hong Kong strain (WHO strain designation A_2_/University of Maryland/1/70) and found that 0%, 57%, and 100% had neutralization titer ≥1∶32 1, 2, and 3 wk after exposure [31]. Our study suggests that serologic follow-up of around 100 cases for 28 d would be sufficient for supporting sero-surveillance.

Our study has several limitations. First, our serologic specimens were collected via convenience sampling of blood donors, hospital outpatients, and vaccine trial participants. As such, our serologic data did not necessarily provide a representative description of pdmH1N1 seroprevalence in the general population. However, our estimates of age-specific IARs were similar to those in a companion serologic study in Hong Kong that was based on paired-sera from households recruited using random digit-dialing of landlines [8]. Second, we assumed that the proportion of pdmH1N1 cases that eventually developed MN titer ≥1∶40 was similar to that observed in serologic follow-up of virologically confirmed cases who reported symptoms [14],[21]. It is not known whether asymptomatic cases were equally likely to develop MN titer, so our estimates of IAR and IHP would need to be revised if new data on this became available. Third, during a pandemic, the reporting delay of clinical surveillance data and the laboratory capacity available for serologic testing are subject to considerable uncertainty. In our model, we assumed that these factors were not the rate-limiting steps for serial cross-sectional sero-surveillance (the number of specimens needed and pre-existing seroprevalence were the primary limiting factors). Finally, we have considered only the serial cross-sectional design for sero-surveillance. An alternative design is cohort-based sero-surveillance, in which sera from the same individuals are collected at various time points during a pandemic, and IARs are inferred from seroconversion rates (i.e., using paired serology) [8]. While the performance of the latter design may have the advantage of being relatively insensitive to pre-existing seroprevalence, it is not obvious how to optimally time the collection of sera from the cohort for real-time surveillance during a pandemic (because regular or frequent blood sampling of the same individuals is unlikely to be feasible). We plan to compare the serial cross-sectional design with the cohort-based design in future studies.

In conclusion, we estimated that if the pre-existing seroprevalence could be adjusted to near zero with around θ = 60%–100% of infected cases reaching seropositivity 6–16 d after symptom onset on average, then serial cross-sectional sero-surveillance with about 300 specimens per week would allow reliable estimates of IHP and IAR as soon as θ × IAR reached around 6% (Figure 5). This level of testing capacity should be logistically feasible for most developed countries if sero-surveillance is a formal part of pandemic surveillance. Once an accurate estimate of IAR is available, reliable estimates for other severity measures such as the probability of intensive care unit admission or death given infection can then be easily obtained. Once reliable severity estimates have been obtained for a high-priority group, testing capacity could then be allocated to other groups. Concentrated efforts to gather such data from one of the major cities affected early in the course of a pandemic would potentially yield data that is of global relevance for public health. Such strategies would be useful not only for situational awareness of influenza pandemics but also for pandemics caused by other pathogens, e.g., a future SARS-like event. As such, serologic surveillance should be considered in updated plans for influenza pandemic preparedness and response and for other pandemics.

## Supporting Information

Figure S1 **Age-specific proportions of individuals with antibody titers ≥1∶40 by viral MN in Hong Kong since June 2009.** Markers and vertical bars indicate the MLE and 95% confidence intervals of weekly seroprevalence estimated using the exact binomial method applied to weekly serologic data. Data points with sample size <10 are not shown. The seroprevalence for 5–14 y olds in early June 2009 was estimated using blood samples collected in April 2009 from the 5- to 14-y-old participants of a pediatric cohort study.
(EPS)

Figure S2 **The number of serum specimens that would have been needed to yield reliable estimates of IHP by mid-August during the 2009 influenza pandemic.** The estimate of IHP in the full model was assumed to be the true IHP (Table 1). Each tick on the *x*-axis indicates the first day of the month. Black circles indicate the median, while boxes and vertical bars indicate the IQRs and 95% confidence intervals of the ordinate.
(EPS)

Figure S3 **The correspondence between θ × IAR and seroprevalence in 1,000 randomly generated epidemic scenarios.**
(EPS)

Text S1 **Detailed study design and preliminary analyses.**
(DOC)

## Abbreviations

IAR: infection attack rate
IHP: infection-hospitalization probability
IQR: interquartile range
MLE: maximum likelihood estimate
MN: microneutralization
WHO: World Health Organization
