# Supplementary material for: Estimating Infection Attack Rates and Severity in Real Time during an Influenza Pandemic: Analysis of Serial Cross-Sectional Serologic Surveillance Data
Source: PLoS Med. 2011 Oct 4;8(10):e1001103. doi: 10.1371/journal.pmed.1001103 (PMC3186812; doi:10.1371/journal.pmed.1001103)
Supplement: Text S1 — Detailed study design and preliminary analyses. (DOC) [file pmed.1001103.s004.doc]

**Estimating infection attack rates and severity in real-time during an influenza pandemic: analysis of serial cross-sectional serologic surveillance data**

Text S1

Joseph T. Wu^1^, Andrew Ho^1^, Edward S. K. Ma^2^, Cheuk Kwong Lee^3^, Daniel K. W. Chu^2^, Po-Lai Ho^2^, Ivan F.N. Hung^4^, Lai Ming Ho^1^, Che Kit Lin^3^, Thomas Tsang^5^, Su-Vui Lo^6,7^, Yu-Lung Lau^8^, Gabriel M. Leung^7^, Benjamin J. Cowling^1^*, J. S. Malik Peiris^2,9^*

1. Department of Community Medicine and School of Public Health, Li Ka Shing Faculty of Medicine, The University of Hong Kong, Hong Kong Special Administrative Region, People’s Republic of China.
2. Department of Microbiology, Li Ka Shing Faculty of Medicine, The University of Hong Kong, Hong Kong Special Administrative Region, People’s Republic of China.
3. Hong Kong Red Cross Blood Transfusion Service, Hospital Authority, Hong Kong Special Administrative Region, People’s Republic of China.
4. Department of Medicine, Li Ka Shing Faculty of Medicine, The University of Hong Kong, Hong Kong Special Administrative Region, People’s Republic of China.
5. Centre for Health Protection, Department of Health, Government of the Hong Kong Special Administrative Region, People’s Republic of China.
6. Hospital Authority, Hong Kong Special Administrative Region, People’s Republic of China.
7. Food and Health Bureau, Government of the Hong Kong Special Administrative Region, People’s Republic of China.
8. Department of Paediatrics and Adolescent Medicine, Li Ka Shing Faculty of Medicine, The University of Hong Kong, Hong Kong Special Administrative Region, People’s Republic of China.
9. HKU-Pasteur Research Center, Hong Kong Special Administrative Region, People’s Republic of China.

Corresponding author and author for reprint requests:

Joseph T. Wu, School of Public Health, Li Ka Shing Faculty of Medicine, The University of Hong Kong, Units 624-7, Cyberport 3, Pokfulam, Hong Kong.

Tel: +852 3906 2009; Fax: +852 3520 1945; email: joewu@hku.hk

**Table of Contents**

[Serologic data 3](#__RefHeading___Toc298975246)

[The generalized algorithm for real-time estimation of infection-hospitalization probability 4](#__RefHeading___Toc298975247)

[Analysis of published data on the kinetics of antibody response against pdmH1N1 among lab-confirmed cases in the United States 7](#__RefHeading___Toc298975248)

[Sample sizes needed to obtain reliable estimates of IHP by mid-August 2009 during the 2009 influenza pandemic 10](#__RefHeading___Toc298975249)

[Correspondence between infection attack rate and seroprevalence during the early phase of an epidemic 11](#__RefHeading___Toc298975250)

# Serologic data

Fig. S1 summarizes the data from our serologic survey of pdmH1N1 in Hong Kong . This figure suggested that seroprevalence were largely similar among the three groups of subjects (blood donors vs. hospital outpatients vs. subjects of a community vaccination study) except for the 15-19 yo after November 2009. The total numbers of serum samples tested were as follows: 13,283 blood donor samples between June 2009 and March 2010, 3,613 hospital outpatient samples between September 2009 and May 2010, and 917 samples from the pediatric study cohort between April 2009 and January 2010.

# The generalized algorithm for real-time estimation of infection-hospitalization probability

Consider a population of size *N* (which can be an age group or risk group). Assume that the infection-hospitalization probability (IHP) is constant over time. Let $n_{t_{i}}$be the number of cross-sectional serum samples for serologic surveillance at time *t_i_*. Let $x_{t_{i}}$ be the number of positive serologic results among the $n_{t_{i}}$ serum samples tested. We assume that the following are empirically observable from pandemic surveillance: (i) the probability distributions of the time from illness onset to hospitalization (cdf *F_Hosp_* and pdf *f_Hosp_*); (ii) the probability distribution of the time from illness onset to seropositivity for those who were seronegative before infection (cdf *F_Seropos_*); and (iii) the proportion of infections that eventually became seropositive *θ*. We assume that the maximal delay from onset to hospitalization is *M* days. The algorithm for real-time estimation of IHP is as follows. At any time *t* during the epidemic:

1. Use *F_Hosp_* to deconvolute the daily hospitalizations time series *h*_0_,…,*h_t_* to obtain an unscaled daily incidence curve *a*_0_,…,*a_t_* . If IHP is known, the true incidence curve is estimated by dividing *a*_0_,…,*a_t_* by IHP. Note that this step can be skipped if the actual onset dates of hospitalized cases are known. This deconvolution step comprises providing an initial estimate for the unscaled incidence curve and then iterating the EM algorithm until the estimated unscaled incidence curve converges. Suppose our estimated unscaled incidence curve is $a_{0}^{n},...,a_{t}^{n}$ after the *n*-th iteration of the algorithm. The corresponding hospitalization curve after the *n*-th iteration is therefore $h_{s}^{n}=\sum_{u=0}^{s} f_{Hosp}(t-u)a_{u}^{n},\text{ }s=0,...,t$. The estimated unscaled incidence curve for the (*n*+1)-th iteration is then obtained using the formula

$$a_{s}^{n+1}=\frac{a_{s}^{n}}{1-F_{Hosp}(s)}\sum_{u=s}^{M} \frac{f_{Hosp}(u-s)h_{u}}{h_{u}^{n}}.$$

The estimated unscaled incidence curve $a_{0}^{n},...,a_{t}^{n}$ typically converges after *Q* = 10 iterations. Note that near the end of the time horizon (i.e. times *t*, *t*–1, *t*–2, etc), the observed numbers of hospitalization do not reflect all the infections occurred at those times. For example, among those infections occurred at time *t*–*s* that would eventually be hospitalized, only those who had ≤*s* days of delay from symptoms onset to hospitalization would be observed in the hospitalization data *h*_0_,…,*h_t_*. To adjust for this right-truncation effect, we make the following adjustment to obtain the unscaled incidence curve $a_{0},...,a_{t}$:

$$a_{s}=\left\{\begin{matrix} a_{s}^{Q} & \text{for }s\leq t-M, \\ \frac{a_{s}^{Q}}{F_{Hosp}(t-s)} & \text{for }s>t-M. \end{matrix}\right.$$

1. Use *F_Seropos_* to construct an estimated seroprevalence curve *b*_0_,…,*b_t_* from the unscaled incidence curve *a*_0_,…,*a_t_*:

$$b_{s}=P_{0}+\sum_{u=0}^{s} \left[\kappa P_{0}+(1-\kappa P_{0})\theta F_{Seropos}(s-u)\right]a_{u},\text{ }s=0,...,t$$

where *P*_0_ is the pre-existing seroprevalence rate and *θ* is the proportion of recovered individuals who eventually became seropositive. This formula assumes that the titer cutoff for seropostivity is such that individuals who are seropositive before the pandemic are *κ* as susceptible to pandemic infections as seronegative individuals. If the titer cutoff is such that either *P*_0_ is close to zero or seropositive individuals are mostly immune to pandemic infection (*κ* is close to zero), then the formula simplifies to:

$b_{s}=P_{0}+\frac{\theta}{IHP}\sum_{u=0}^{s} F_{Seropos}(s-u)a_{u},\text{ }s=0,...,t$.

We used this formula in the main text because the prepandemic seroprevalence was sufficiently low in our pdmH1N1 data (<5% with MN titer 1:40 as the cutoff) and our simulations and *θ* was interpreted (accurately) as the proportion of pandemic infections who eventually became seropositive.

1. Find the best values of IHP and *P*_0_ for fitting the estimated seroprevalence curve *b*_0_,…,*b_t_* to the serial cross-sectional serologic data using the following likelihood function:

where the product is over all times *t_i_* ≤ *t* at which cross-sectional serologic data are available with

- *Binomial*(*x*, *n*, *p*) is the binomial probability of getting *x* seropositives from *n* tests if the true seroprevalence rate is *p*:

- $n_{t_{i}}$ is the number of serum samples tested at time *t_i_* and $x_{t_{i}}$ is the number of these samples that are seropositive.
- *q_sensitivity_* and *q_specificity_* are the sensitivity and specificity of the serologic test, which should be readily known from laboratory surveillance.

The 95% credible intervals of IHP estimate can be obtained using profile-likelihood. IAR at time *t* can then be obtained by dividing the unscaled infection attack rate $\sum_{s=0}^{t} a_{s}$ by the estimated IHP.

# Analysis of published data on the kinetics of antibody response against pdmH1N1 among lab-confirmed cases in the United States

We used the data published in Veguilla et al. to estimate the kinetics parameters of antibody response against pdmH1N1. However, our definition of MN titer is slightly different from theirs. By convention, MN titers were previously denoted by taking into account the final dilution resulting from mixing the serum dilution with the virus. The WHO Manual for laboratory diagnosis recommends that the virus titer is now denoted as the initial serum dilution alone . While our previous publication and the current study used the old definition, the recent paper by Veguilla et al. used the new convention. In effect, our MN titers (in and the current study) need to be halved when comparing with those of Veguilla et al. . We have incorporated this normalization in our current study when estimating the kinetics parameters as follows.

The following pdmH1N1 antibody response data were extracted from Table 2 and Figure 3 in Veguilla et al. :

| Days since symptoms onset when serum was collected | 0* | ≤ 7 | 8-14 | 15-21 | >21 |
| --- | --- | --- | --- | --- | --- |
| No. of sera tested, *n_i_* | 127 | 46 | 23 | 21 | 72 |
| No. of sera with MN titer ≥1:40 (MN titer ≥1:20 in ), *x_i_* | 23 | 8 | 17 | 21 | 72 |
| Percentage of sera seropositive | 18% | 17% | 74% | 100% | 100% |
| Assumed collection time (days since symptoms onset), *t_i_* | 0 | 4 | 11 | 18 | ∞ |

* Approximated using sera from non-exposed 0-39 yo individuals in the study

We used MN titer 1:40 as the cutoff for seropositivity. The results in Veguilla et al. suggested that our MN titer 1:40 (their MN titer 1:20) corresponded to an HI titer 1:10 which would unlikely provide substantial protection against pandemic infection (because, as mentioned in their paper, an HI titer of 1:40 has generally been associated with 50% reduction in the risk of influenza illness ). As such, when estimating the kinetics parameter from their data, we assumed that individuals who were seropositive before the pandemic were as susceptible to pandemic infections as seronegative individuals (i.e. *κ* = 1 in Step 2 of the previous section). Therefore, the proportion of cases seropositive on 0 day post symptoms onset, *ρ*_0_, could be estimated by the proportion of 0-39 yo who were not exposed to pdmH1N1 but were seropositive (in the raw data in Table 2 of Veguilla et al , *x*_0_ = 23 out of *n*_0_ = 127 non-exposed 0-39 yo individuals were seropositive). We assumed that among infected cases who were seronegative before infection, a proportion *θ* of them would eventually become seropositive and the time between symptoms onset and seropositivity followed an Erlang-10 distribution *F_Seropos_* with mean *μ_Seropos_*. Because the exact times of serum collection were not published in Veguilla et al , we assumed that for sera collected ≤ 21 days after symptoms onset, the collection times were the midpoints of the corresponding collection time intervals, i.e. 4 , 11, and 18 days, respectively. For those sera collected >21 days after symptoms onset, we assigned ∞ to be their collection times, i.e. we assumed that the values of *F_Seropos_* at these collection times were 1*.* Under these assumptions, the expected proportion of sera that were seropositive during the *i*th time interval (*t_i_*) was

$$\rho_{i}=\rho_{0}+\theta(1-\rho_{0})F_{Seropos}(\mu_{Seropos},t_{i})$$

where *F_Seropos_*(*μ_Seropos_*, *t*) was the probability that the time between symptoms onset and seropositivity (among individuals who were seronegative before infection) was smaller than *t* given that the mean was *μ_Seropos._*. We could then construct the following likelihood for these antibody response data:

where *Binomial*(*x*, *n*, *ρ*) was the binomial probability of getting *x* seropositives from *n* sera if the true seropositivity rate was *ρ* and the product was over the collection time intervals*.* Using Monte Carlo Markov Chain with non-informative (i.e. flat) priors for all parameters, the posterior mode (which was the same as the MLE in the classical framework) was *θ* = 1 (95% credible interval, 0.95-1) and *μ_Seropos_* = 9.6 (8-11.5) days after 5,000,000 iterations. The posterior distributions are shown in Fig. 2B-C. Note that the notational dependence of *L*_A_ on *ρ*_0_ was dropped in the main text for conciseness.

We performed sensitivity analyses to ensure that our results were robust against the assumptions we made when estimating the kinetics parameters. First, if the seropositivity rate on 0 day post symptoms onset was taken as 0% instead of the 18% among non-exposed 0-39 yo (this is equivalent to assuming that the sera in Figure 3 of Veguilla et al. were all from cases who were seronegative before infection), the kinetics would be faster with *μ_Seropos_* = 8 days which would improve the performance of sero-surveillance. To make a conservative assessment of the performance of sero-surveillance, we did not use this assumption. Second, we repeated all analyses in the main text using Erlang distributions with 5, 20 and 40 stages for *F_Seropos._*. The posterior modes and 95% credible intervals are shown in the following table.

|  | *Θ* | *μ_Feropos_* (days) |
| --- | --- | --- |
| Erlang-5 | 1 (0.95-1) | 9.3 (7.5-11.5) |
| Erlang-10 | 1 (0.95-1) | 9.6 (8-11.5) |
| Erlang-20 | 1 (0.95-1) | 10 (8.7-11.5) |
| Erlang-40 | 1 (0.95-1) | 10.3 (9.3-11.4) |

All results remained almost the same because the posterior distribution for *θ*  and *μ_Seropos_* were very similar for all the Erlang distributions considered.

# Sample sizes needed to obtain reliable estimates of IHP by mid-August 2009 during the 2009 influenza pandemic

To estimate the number of specimens that would have been needed in order to obtain reliable estimates of IHP for pdmH1N1 by mid-August 2009 (4 weeks before the epidemic peak), we assumed that the incidence and seroprevalence curves in the full model were accurate. We simulated 300 stochastic realizations of serial cross-sectional sero-surveillance in which (i) *m* pre-pandemic specimens were used to estimate seroprevalence on June 30 and (ii) *m* specimens were collected and tested every week starting in the 4^th^ week of July. We searched for the smallest value of *m* for each age group that would have yielded reliable estimates of IHP by mid-August. Fig. S2 shows that we would have needed around 150, 350 and 500 specimens per week for 5-14 yo, 15-19 yo and 20-29 yo in order to obtain reliable estimates of IHP for these age groups by mid-August. For the 30-59 yo, even with a prohibitively large sample size of 800 per week, reliable estimates of IHP would not have been available until mid- to late-September because of the very low ratio of IAR to pre-existing seroprevalence for these age groups.

# Correspondence between infection attack rate and seroprevalence during the early phase of an epidemic

To show that the correspondence between *θ* × IAR and seroprevalence was robust against epidemic model structure and parameter values, we simulated 1,000 epidemics scenarios that were randomly generated using Latin-hypercube sampling of the parameter space considered in Fig. 5. In each scenario, we recorded the value of *θ* × IAR when seroprevalence was 0.5%, 1%, 2% and 3%. We plotted these values against the ratio of epidemic doubling time to the mean time between illness onset and seropositivity which we denoted by *α* (Fig. S3). The results showed that the correspondence between IAR and seroprevalence depended on the epidemic model only via *θ* and *α*.

**References**

1. Wu JT, Ma ESK, Lee CK, Chu DKW, Ho PL, et al. (2010) The infection attack rate and severity of 2009 pandemic influenza (H1N1) in Hong Kong. Clinical Infectious Diseases 51: 1184-1191.

2. Goldstein E, Dushoff J, Ma J, Plotkin JB, Earn DJ, et al. (2009) Reconstructing influenza incidence by deconvolution of daily mortality time series. Proc Natl Acad Sci U S A 106: 21825-21829.

3. Cauchemez S, Boelle PY, Donnelly CA, Ferguson NM, Thomas G, et al. (2006) Real-time estimates in early detection of SARS. Emerg Infect Dis 12: 110-113.

4. Veguilla V, Hancock K, Schiffer J, Gargiullo P, Lu X, et al. (2011) Sensitivity and Specificity of Serologic Assays for Detection of Human Infection with 2009 Pandemic H1N1 Virus in U.S. Populations. J Clin Microbiol 49: 2210-2215.

5. WHO Global Influenza Surveillance Network (2011) Manual for the laboratory diagnosis and virological surveillance of influenza. Malta: World Health Organization.

6. Hobson D, Curry RL, Beare AS, Ward-Gardner A (1972) The role of serum haemagglutination-inhibiting antibody in protection against challenge infection with influenza A2 and B viruses. J Hyg (Lond) 70: 767-777.
